# Effects of Huolisu Oral Solution on Depression-Like Behavior in Rats: Neurotransmitter and HPA Axis

**DOI:** 10.3389/fphar.2022.893283

**Published:** 2022-06-02

**Authors:** Min Xiao, Kaiyong Xie, Li Yuan, Jun Wang, Xing Liu, Zhonghua Chen

**Affiliations:** ^1^ Department of Pharmacy, West China Hospital, Sichuan University, Chengdu, China; ^2^ Chengdu Di’ao Group Tianfu Pharmaceutical Group Co., Ltd., Chengdu, China; ^3^ Department of TCM Pharmacy, Chengdu Integrated TCM&Western Medicine Hospital, Chengdu, China; ^4^ Department of Orthopaedic Surgery, The Children’s Hospital Chongqing Medical University, Chongqing, China; ^5^ Key Laboratory of Drug-Targeting and Drug Delivery System of the Education Ministry and Sichuan Province, Sichuan Engineering Laboratory for Plant-Sourced Drug and Sichuan Research Center for Drug Precision Industrial Technology, West China School of Pharmacy, Sichuan University, Chongqing, China

**Keywords:** HLS, antidepressant, CUMS, neurotransmitter, HPA axis

## Abstract

**Background:** Depression is a common mental disorder, and its morbidity rate is expected to rank second among all mental disorders by 2020. Hence, traditional Chinese medicines gradually attract the attention of many researchers because of their various targets and low toxicity. Huolisu oral solution (HLS) is a Chinese medicine compound preparation, which is present in the Chinese Pharmacopoeia. It is used clinically mainly for the treatment of neurasthenia, perimenopausal syndrome, and insomnia, or depression associated with cerebrovascular disease. Despite the fact that HLS has been used as an antidepressant in clinics, the underlying mechanism is still an untouched domain. To provide a theoretical basis for the clinical application, a series of assessment methods, such as the tail suspension test (TST), forced swim test (FST), and locomotor activity test in mice and rat models of chronic unpredictable mild stress (CUMS), have been conducted in our study.

**Objective:** The aim of the study was to explore the antidepressive effect and mechanism of HLS.

**Methods:** CUMS was induced in rats to simulate a depression-like behavior. Neurotransmitters and hormones were detected by enzyme-link immunosorbent assay (ELISA). Pathomorphology examination of the hippocampus was obtained by using the TSView 7 image analysis system. The active ingredients of HLS were also determined by high-performance liquid chromatography (HPLC).

**Results:** HLS could alleviate the depression-like behavior of the model rats. Biochemical analysis showed that HLS enhanced the levels of 5-hydroxytryptamine (5-HT), norepinephrine (NE), and dopamine (DA) in the hippocampus and diminished these in the serum of the CUMS rats. HLS could also decrease the concentration of corticosterone (CORT), adrenocorticotropic hormone (ACTH), and β-endorphin (β-EP) in blood. The pathohistological examination revealed that the hippocampus and adrenal gland were improved after treatment with HLS.

**Conclusions:** This study concluded that HLS could alleviate depression-like behaviors in the rats exposed to CUMS, and the potential mechanism may be related to the regulation of the monoamine neurotransmitters, the hypothalamic–pituitary–adrenal (HPA) axis, and the β-EP. These findings hint that HLS is likely to be a potentially effective agent for treating depression.

## Introduction

Depression is a common mental disorder, and its morbidity rate is expected to rank second among all mental disorders by 2020 (Luo et al., 2012). Although the pathogenesis of depression is still controversial, it is believed that depression may be the result of an interaction of many factors including psychosocial factors and various biological changes (Liu et al., 2013). The major psychosocial factors include intense work and fast-paced life, bad interpersonal relationships, and sudden emotional stress. Hypotheses related to the biochemical mechanism of depression mainly include the monoamine neurotransmitter hypothesis, receptor hypothesis, endocrine hypothesis, and immune change hypothesis (He et al., 2006; Liu B. et al., 2017; Jesulola et al., 2018). Based on the monoamine hypothesis, the level of various monoamine neurotransmitters, such as norepinephrine (NE), dopamine (DA), and 5-hydroxytryptamine (5-HT), in the central system is reduced in patients with depression (Anisman et al., 2008; Hamon and Blier, 2013; Leonard, 2014). Based on the receptor hypothesis, depression results from increased sensitivity (hypersensitivity) of NE/5-HT receptors in the brain of patients are observed. The endocrine hypothesis involves the hypothalamic–pituitary–adrenal (HPA) axis (Anacker et al., 2011). On being exposed to stress conditions, the level of corticotropin-releasing hormone (CRH), adrenocorticotropic hormone (ACTH), and glucocorticoids (GC) will be enhanced because of the hyperfunction of the HPA axis. To worsen, the immune responses will be inhibited (Belvederi Murri et al., 2014; Lange et al., 2013; O'Keane et al., 2012).

At present, the chemical medicines take up the main part of the clinical antidepressants based on the different hypothesis of pathogeny, but the defects, such as narrow spectrum, untoward effect, and high price, limit their long-term applications (Xu et al., 2005). Hence, traditional Chinese medicines gradually attract the attention of many researchers because of their various targets and low toxicity (Gao et al., 2013; Wang et al., 2017). Huolisu oral solution (HLS) is a Chinese medicine compound preparation, which is present in the Chinese Pharmacopoeia (National Pharmacopoeia Committee, 2010). It consists of processed *Reynoutria multiflora* (Thunb.) Moldenke [Polygonaceae; Polygoni Multiflori Radix Praeparata], *Epimedium brevicornu* Maxim. [Berberidaceae; Epimedii Folium], *Polygonatum sibiricum* F. Delaroche [Asparagaceae; Polygonati Rhizoma], *Lycium chinense* Mill. [Solanaceae; Lycii Fructus], *Astragalus mongholicus* Bunge [Leguminose; Astragali Radix], and *Salvia miltiorrhiza* Bunge [Lamiaceae; Salviae Miltiorrhizae Radix et Rhizoma], and the main effects of HLS include invigorating Qi and enriching blood, nourishing the liver and kidney. It is used clinically mainly for the treatment of neurasthenia, perimenopausal syndrome, and insomnia or depression associated with cerebrovascular disease (Zhan et al., 2008). Despite the fact that HLS has been used as an antidepressant in clinics, the underlying mechanism is still an untouched domain. To provide a theoretical basis for the clinical application, a series of assessment methods, such as TST, FST, locomotor activity test in mice and rat models of CUMS, have been conducted in our study. Based on the monoamine hypothesis and endocrine hypothesis, we investigated 5-HT, DA, NE and ACTH, corticosterone (CORT), and β-endorphin (β-EP) by ELISA. In addition, we measured the content of the major antidepressive ingredients in the recipes according to the published literature, thus confirming the antidepressive effect of HLS from another point of view.

## Materials and Methods

### Animals

In total, 100 male Wistar rats of SPF grade aged 7–8 weeks old, weighing 200 ± 20 g, were provided by Chengdu Dashuo Biological Technology Co., Ltd; the certification number is SCXK (Chuan) 2013-24. A total of 108 Kunming mice of SPF grade aged 6–8 weeks old, weighing 24–29 g, were provided by the Laboratory Animal Center of Sichuan University (certification No.: SCXK (Chuan) 2014-09). The animals were kept in constant environmental conditions (22–26°C, relative humidity 50–60%, 12 h light/dark cycle) and food and water were made available *ad libitum* (except during the period of CUMS). The experimental protocol was approved by the Ethical Review Committee of Society for Laboratory Automation and Screening (SLAS), and all the animal procedures were approved by the Institutional Animal Care and Use Committee.

### Drugs and Reagents

HLS (160402) was provided by Chengdu Di’ao Group Tianfu Pharmaceutical Co., Ltd. The procedure of HLS is shown in Supplementary Material. Sertraline hydrochloride tablets (150801) were purchased from Pfizer Pharmaceutical Limited (Dalian, China) and prepared as 37.5 and 62.5% suspensions in 0.5% CMC-Na prior to use. ELISA kits (201504), 5-HT, NE, DA, ACTH, CORT, and β-EP, were provided by Shanghai Fengxiang Biological Technology Co., Ltd. PBS, chloral hydrate, and paraformaldehyde of analytic grade were also used.

Icariin standard substance (110737–201415), astragaloside IV standard substance (0781–200311), and diosgenin standard substance (111539–201001) were provided by the National Institute for the Control of Pharmaceutical and Biological Products. 2,3,5,4′-Tetrahydroxy stilbene-2-Ο-β-D-glucoside standard substance (110844–201109) and tanshinone IIA standard substance (110766–201519) were provided by the National Institute for Food and Drug Control. N-butanol, absolute methanol, and ammonia water of analytic grade were also used.

Negative control substances for processed polygoni multiflori radix praeparata, epimedii folium, polygonati rhizoma, astragali radix, and salviae miltiorrhizae radix et rhizoma were provided by Chengdu Di’ao Group Tianfu Pharmaceutical Co., Ltd.

### Main Instruments

The ZZ-6 apparatus was used for measuring the mouse locomotor activity (Chendu Taimeng Science and Technology Co., Ltd.); self-made open field box, 250Q rat holder, MK3 microplate reader (Thermo Fisher, United States), Allegra X-22R centrifuge (Beckman, United States), 81m-25 optical microscope (Olympus), TH-86-340-WA ultra-low temperature freezer (−80°C, Germany), high-performance liquid chromatography system (Shimadzu LC-6AD), evaporative light scattering detector (SEDEX75), ultraviolet spectrophotometer (Alpha-1860), Sartorius electronic analytical balance 0.0001 g (Germany), and electronic analytical balance 0.001 g (Germany) were also used.

### Evaluating the HLS With TST, FST, and Locomotor Activity Test in Mice

#### Groups and Drug Administration

In total, 36 mice were randomly divided into three groups with 12 mice in each group, including a normal group that was given 0.5% CMC-Na (20 ml/kg/d), a positive control group which was treated with sertraline (5 mg/kg/d), and HLS group which was then treated with HLS (20 ml/kg/d, 2.7 g crude medicine per ml). The corresponding drugs were intragastrically administered for 7 days.

#### Tail Suspension Test (TST)

The TST was performed, as previously described (Cates et al., 2013). Mice were individually suspended by the tail with a clamp placed 1–2 cm from the tip of the end. After 1 min of adaptation, the immobility time of the animals was recorded in the subsequent 5 min.

#### Forced Swim Test (FST)

The FST employed was similar to that described in a previous report (Hodosy et al., 2012). Mice were placed in a rectangular white tank (20 × 10 × 13 cm^3^) filled with water at 25 ± 1°C. After forced swimming for 6 min, immobility time was recorded in the subsequent 5 min. After each trial, the apparatus was cleaned.

#### Locomotor Activity Test

Mice were placed in a ZZ-6 apparatus for measuring locomotor activity in a quiet environment. After 3 min of adaptation, the locomotor activity of the animals was recorded in the subsequent 5 min.

### Anti-Depressive Effects of HLS and the Underlying Mechanisms

#### Groups and Drug Administration

**FIGURE 1:**
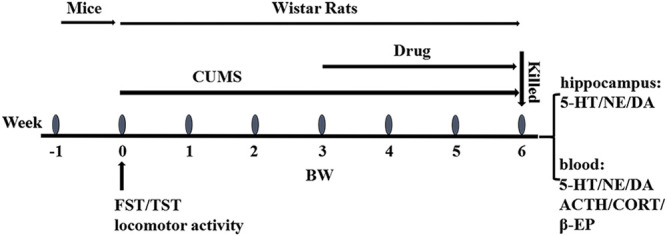
Overall flow of the experimental design: FST, TST, and locomotor activity were conducted in mice for preliminary screening and evaluation in 1 week. CUMS was inducted into rats from week 0 to week 6. The drugs were administrated at week 3 and lasted 3 weeks. The body weight and the open field score were obtained at weeks 0, 3, 4, 5, and 6. The animals were sacrificed for biological analysis of the hippocampus and blood at week 6.

A total of 100 male Wistar rats underwent the open field test for behavioral scoring. The rats with scores between 30 and 120 were divided into four groups using a randomized block design: group 1 which was a normal group (normally reared rats, 20 ml/kg/d), group 2 which was a model group (depressive rats without treatment), group 3 which was a positive control group (depressive rats treated with sertraline, 5 mg/kg/d), and group 4 which was an HLS group (depressive rats treated with HLS, 20 ml/kg/d, and 2.7 g crude medicine per ml). After successful induction of depression by CUMS and separation for 21 days in groups 2, 3, and 4, the rats were intragastrically given the corresponding drugs for 21 days, except for the model group without treatment. During the treatment period, CUMS and separation were still administered. The experimental design can be seen in Figure 1.

#### Induction of Depression by CUMS and Separation

The CUMS model was revised from a report on models of depression that allow investigation of key biological correlates of depression (Nollet et al., 2013). Except for group 1 rats, which were normally reared (5–6 rats/cage), the others were reared individually in a separate cage and given two different stimuli randomly daily; each stimulus was administered four times in 21 days. The stimuli included swimming in cold water at 4°C for 5 min, thermal stimulation (hot plate test, at 45°C, for 5 min), water deprivation (24 h; no fasting), fasting (24 h; no water deprivation), day/night reversal (24 h), moist bedding (10 h), restraint (1 h), and vibration (1 min).

#### Open Field Test (OFT)

OFT paradigm can be found in the previous report (Aboul-Fotouh, 2015). The rats were put into the central square in an apparatus (100 × 100 × 40 cm^3^) with a black inner side and white lines on the bottom (five in a row and five in line, equally). Then, they were allowed to move freely for 3 min, recording the number of crossings (at least three paws in one square) and rearings (standing upright on the hind legs) and finally calculating the summation.

#### Indicator Measurement

The experimental indicators were measured 1 h after drug administration.

#### Open Field Test and Weight Measurement

Open field tests and weight measurements were performed once a week after drug administration.

#### Measurement of Major Organ Coefficients

One hour after drug administration on day 21st, eight rats of each group were anesthetized (at 8:00–10:30) by intraperitoneal injection of 10% chloral hydrate (0.3 ml/100 g) and then killed by femoral artery bleeding. The bilateral hippocampus, adrenal glands, thymus, and spleen were removed and weighed to calculate the organ coefficients.

#### Determination of the Content of 5-HT, NE, and DA in Hippocampus and Serum

The blood samples were collected, as described earlier, standing for 1 h and centrifuged at 3000 r/min for 20 min, and the supernatants were collected for further use. Hippocampal samples were homogenized in PBS (1:1, w/v, mg/ml) and centrifuged at 3000 r/min for 20 min, and the supernatants were collected and preserved at −80°C for further use. The contents of 5-HT, NE, and DA in the hippocampus and serum were determined using commercial kits, according to the manufacturer’s instructions.

#### Determination of the Content of ACTH, CORT, and β-EP in Serum

The serum contents of ACTH, CORT, and β-EP were determined using commercial kits, according to the manufacturer’s instructions.

#### Pathomorphological Examination

The remaining eight rats of each group were anesthetized by intraperitoneal injection of 10% chloral hydrate (0.3 ml/100 g), fixed in supine position, and perfused with 100 ml normal saline *via* the left ventricle; then, 4% paraformaldehyde was perfused after making an incision on the liver. The hippocampus and adrenal glands were then obtained and weighed. The samples were fixed in 4% paraformaldehyde, embedded in paraffin, and sectioned. One section was selected from every 10 intermittent sections, and five sections were selected for each hippocampus or adrenal gland for hematoxylin and eosin (HE) staining. The TSView7 image analysis system (TUCSEN Imaging Technology, Inc.) was used to count the total number of neurons and the number of normal neurons in five grid squares in the CA3 area of the hippocampus (magnification, ×400) to calculate the proportion of normal neurons. The maximum thickness of the adrenal cortex was measured at a magnification of ×40.

### Determination of the Contents in Active Ingredients

#### Determine the Content of 2,3,5,4′-Tetrahydroxy Stilbene 2-O-β-D-Glucoside

A total of 1 ml of HLS was precisely weighed and placed in a 25 ml volumetric flask. Methanol was added to the 25 ml volume mark. About 2 ml of the obtained solution was sucked in a 10 ml volumetric flask, and methanol was added to the 10 ml volume mark to obtain the test solution of HLS. The contents of the volume bottle were filtered through a 0.45 μm microporous membrane. Methanol was added to 2,3,5,4′-tetrahydroxy stilbene-2-Ο-β-D-glucoside precisely weighed to obtain a standard solution in the concentration of 41.76 μg/ml. A negative sample of HLS without polygoni multiflora radix praeparata was prepared according to the treatment method of the test solution. HPLC was conducted using a Reloasil C18 column (150 mm × 4.6 mm, 5 μm) with a mobile phase of acetonitrile water (22:78) at a velocity of 1 ml/min and a detection wavelength of 320 nm.

#### Determine the Content of Icariin

A total of 1 ml of HLS was precisely weighed and placed in a 25-ml volumetric flask. Methanol was added to the 25 ml volume mark to obtain the test solution. The contents of the volume bottle were filtered through a 0.45 μm microporous membrane. Methanol was added to icariin precisely weighed to obtain a standard solution in the concentration of 39.04 μg/ml. A negative sample of HLS without epimedii folium was prepared according to the treatment method of the test solution. HPLC was conducted by using a Reliasil C18 column (150 mm × 4.6 mm, 5 μm) with a mobile phase of acetonitrile water (30:70) at a velocity of 1 ml/min and a detection wavelength of 270 nm.

#### Determine the Content of Saponins of Rhizoma Polygonati

A total of 1 ml of HLS was precisely measured, ultrasonically extracted in the 80% ethanol solution at 60°C, and the filtered solution was obtained and evaporated to dryness. The residue was dissolved in 1% sodium hydroxide and extracted with water-saturated n-butanol; then the organic solution was mixed together and evaporated to dryness, and the residue was dissolved in methanol to obtain the test solution (1 ml HLS was finally diluted in 10 ml methanol). Methanol was added to diosgenin precisely weighed to obtain a standard solution in the concentration of 39.04 μg/ml. The diosgenin standard solution was absorbed in perchloric acid to prepare a linear series solution of 7.2, 12.0, 16.8, 18, and 26.4 µg/ml; the solutions were placed in a water bath at 65°C for coloration and then placed in an ice bath for cooling. Using the blank solution as a control, absorbance at 410 nm was measured. Linear regression was performed with the absorbance as ordinate and diosgenin content as abscissa, and the regression equation was calculated.

#### Determine the Content of Astragaloside IV

A total of 50 ml of HLS was precisely weighed and extracted with n-butanol. The mixed extracted solution was washed with ammonia and evaporated to dryness. Methanol was added to the residue in a 10 ml volume mark to obtain the test solution. The contents of the volume bottle were filtered through a 0.45 μm microporous membrane. Methanol was added to astragaloside IV precisely weighed to obtain a standard solution in the concentration of 552 μg/ml. A negative sample of HLS without astragali radix was prepared according to the treatment method of the test solution. HPLC was conducted by using a Reloasil C18 column (250 mm × 4.6 mm, 5 μm) with a mobile phase of acetonitrile water (35:65) at a velocity of 1 ml/min. The drift tube temperature of ELSD was set at 40°C, and the gas flow was 1.5 L/min.

#### Determine the Content of Tanshinone ⅡA

A total of 50 ml of HLS was precisely weighed and extracted with ether. The mixed extract was evaporated to dryness. Methanol was added to the residue at a 5 ml volume mark. The contents of the volume bottle were filtered through a 0.45 μm microporous membrane. Methanol was added to tanshinone ⅡA precisely weighed to obtain a standard solution in the concentration of 0.6 μg/ml. A negative sample of HLS without salviae miltiorrhizae radix et rhizoma was prepared according to the treatment method of the test solution. HPLC was conducted by using a Reliasil C18 column (150 mm × 4.6 mm, 5 μm) with a mobile phase of acetonitrile water (22:78) at a velocity of 1 ml/min and a detection wavelength of 320 nm.

### Statistical Analysis

Numerical data are expressed as mean ± standard deviation (SD). SPSS 17.0 software was used to conduct a one-way analysis of variance, followed by an LSD test (when *p* > 0.05) or Dunnett T3 test (when *p* < 0.05). *p* < 0.05 is considered statistically significant. * indicates *p* < 0.05 compared with the normal group, and ^#^ indicates *p* < 0.05 compared with the model group.

## Results

### Basic Components of HLS That Have Antidepressive Effects

#### Effects of HLS on Immobility Times in the Tail Suspension Test and Forced Swim Test

**FIGURE 2:**
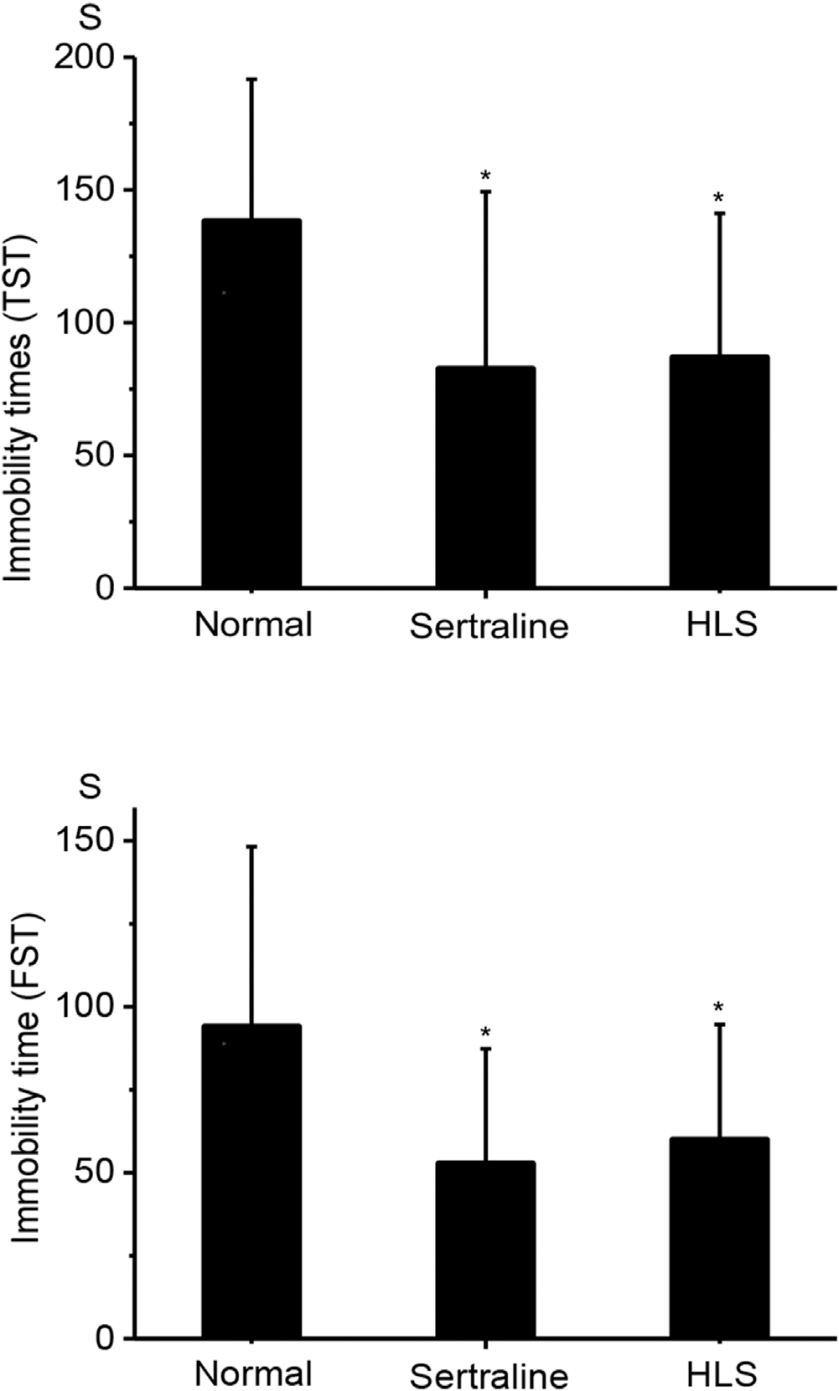
Effects of HLS on the immobility time in TST and FST in mice (‾x ± s, *n* = 12). TST, tail suspension test; FST, forced swimming test. *p < *0.05* vs. normal control.

As shown in Figure 2, HLS could significantly shorten the immobility time in TST and FST compared with the normal group (*p* < 0.05).

#### Effects of HLS on the Mouse Locomotor Activity

**FIGURE 3:**
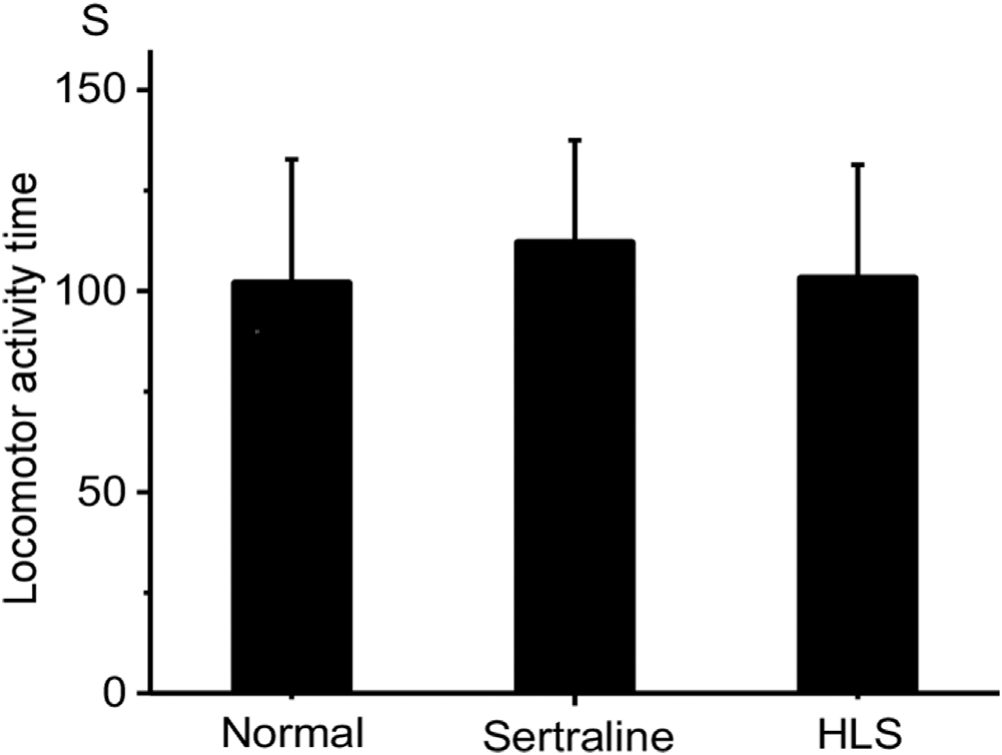
Effect of HLS on locomotor activity in mice (‾x ± s, *n* = 12).

As shown in Figure 3, there was no significant difference in mouse locomotor activity in the HLS group compared with the normal group (*p* > 0.05).

### Effects of HLS on Various Indicators in Depressive Rats

#### Effects of HLS on Open Field Score and Bodyweight

Tables 1, 2 show the effects of HLS on the open field score (OFS) and body weight (BW) on depression-like behavior in rats, respectively. After inducing depression by CUMS and separation, the OFS and BW of the model group were significantly decreased compared with the normal group (*p* < 0.05). It hints that the behavior of rats in the model group is similar to depression largely. HLS or sertraline significantly enhanced OFS after 3-week treatment and increased BW after 1-week treatment compared with the model group (*p* < 0.05).

**TABLE 1:** Effect of HLS on open field scores in rats (‾x ± s, *n* = 16).

Group	Open field score		
	1 week after treatment	2 weeks	3 weeks
Normal	36.71 ± 27.05	46.43 ± 16.74	34.79 ± 18.05
Model	16.00 ± 18.35^a^	25.93 ± 28.69^a^	18.88 ± 21.59^a^
Sertraline	19.53 ± 14.54	48.57 ± 29.68^b^	34.00 ± 26.62^b^
HLS	21.86 ± 12.93	41.50 ± 20.16	34.44 ± 23.88^b^

a *p* < 0.05 vs. normal control.

b *p* < 0.05 vs. model.

**TABLE 2:** Effects of HLS on body weight changes (‾x ± s, *n* = 16).

Group	Pretreatment (g)	1 week after treatment (g)	2 weeks (g)	3 weeks (g)
Normal	310.20 ± 18.42	18.6 ± 7.8	34.8 ± 15.3	52.4 ± 21.8
Model	264.23 ± 27.35^a^	6.2 ± 2.4^a^	13.4 ± 4.8^a^	20.8 ± 6.7^a^
Sertraline	260.83 ± 19.54^a^	16.6 ± 6.2^b^	31.4 ± 12.5^b^	48.2 ± 17.8^b^
HLS	262.47 ± 33.36^a^	15.8 ± 5.6^b^	29.6 ± 11.4^b^	49.7 ± 19.3^b^

a *p* < 0.05 vs. normal control.

b *p* < 0.05 vs. model.

#### Effects of HLS on the Monoamine Neurotransmitter Level in the Hippocampus and Serum

**FIGURE 4:**
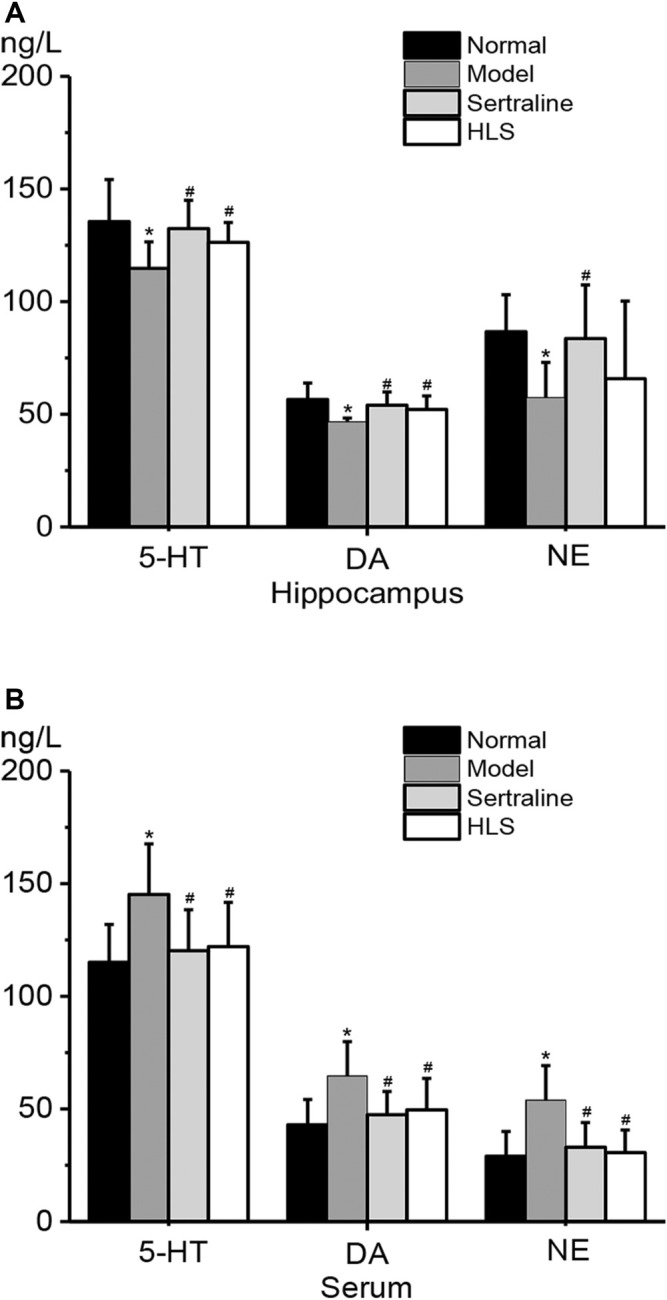
Effects of HLS on the contents of 5-HT, DA, and NE in the hippocampus and serum of rats (‾x ± s, *n* = 8). *p < 0.05 vs. normal control and ^*#*^ *p* < 0.05 vs. model. **(A)** Contents of 5-HT, DA, and NE in the hippocampus. **(B)** Contents of 5-HT, DA, and NE in the serum.

As shown in Figure 4 **,** compared with the normal group, monoamine neurotransmitter levels in the hippocampus were significantly decreased (the *p* < 0.05), but their levels in serum were significantly increased in the model group. Compared with the model group, 5-HT and DA levels in the hippocampus of the HLS group were all significantly increased (*p* < 0.05), but in serum, monoamine neurotransmitter levels were significantly decreased (*p* < 0.05).

#### Effects of HLS on ACTH, CORT, and β-EP Levels in Serum

**FIGURE 5:**
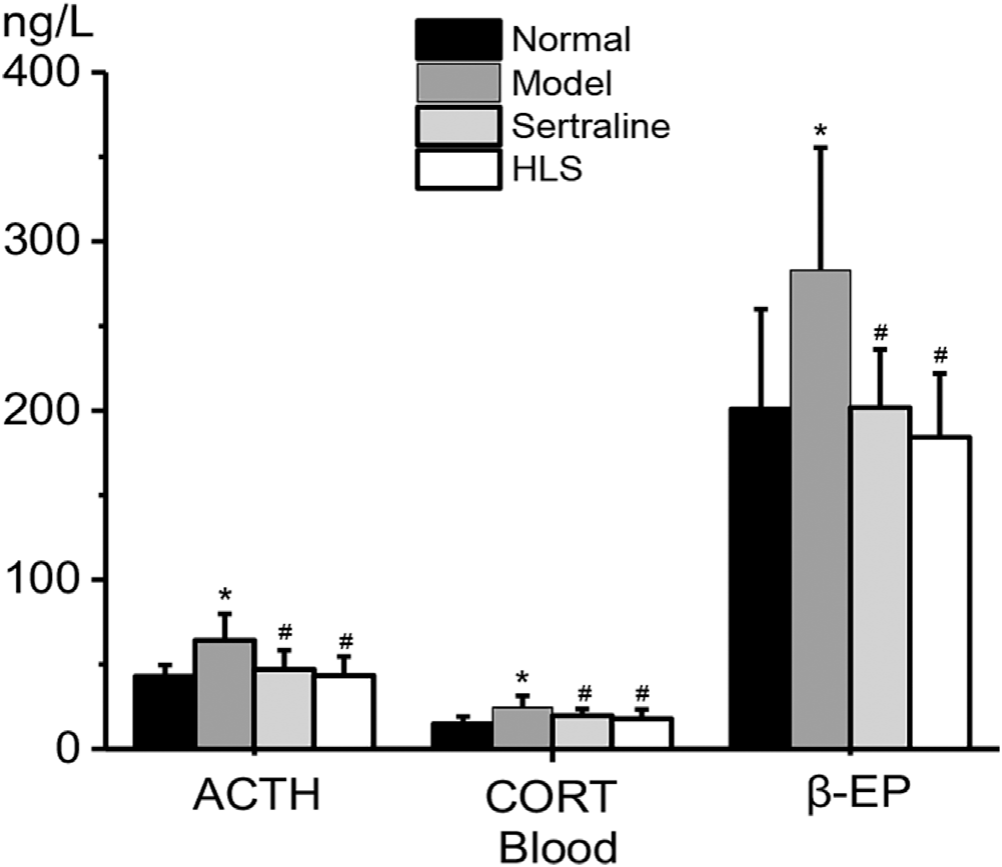
Effects of HLS on the levels of ACTH, CORT, and β-EP in the blood of rats (‾x ± s, *n* = 8). *p < 0.05 vs. normal control and ^*#*^ *p* < 0.05 vs. model.

As shown in Figure 5, ACTH, CORT, and β-EP levels in serum were all significantly higher in the model group than in the normal group (*p* < 0.05). The treatment with HLS significantly decreased ACTH, CORT, and β-EP levels in serum (*p* < 0.05).

#### Effects of HLS on Major Organ Coefficients

**FIGURE 6:**
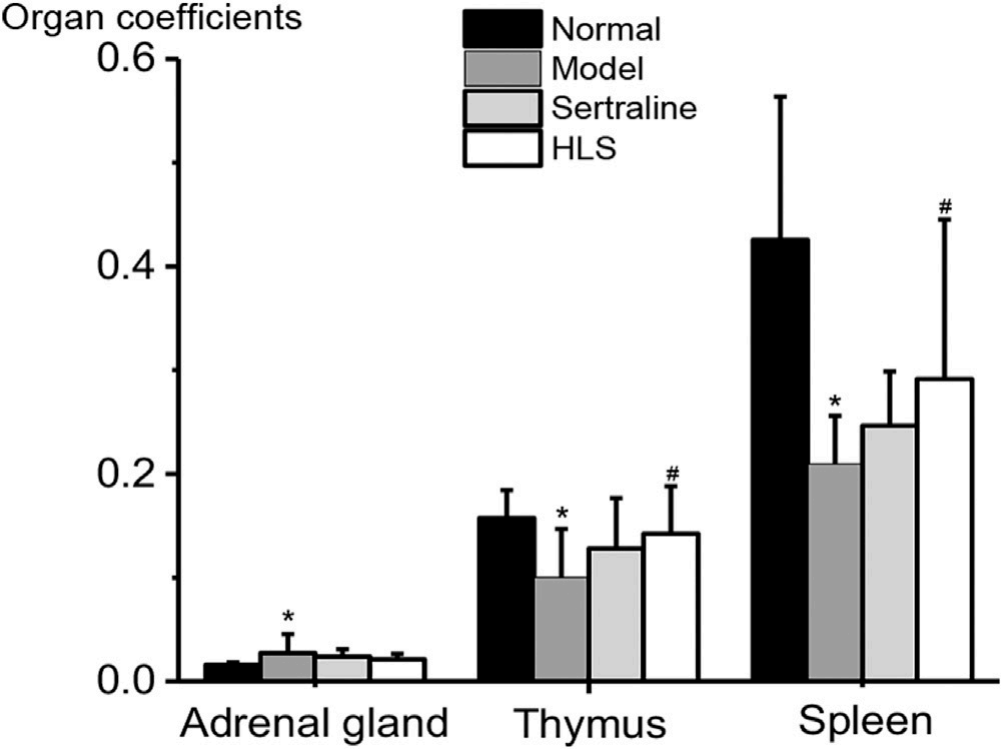
Effects of HLS on organ coefficients in rats (‾x ± s, *n* = 16). *p < 0.05 vs. normal control and ^*#*^ *p* < 0.05 vs. model.

As shown in Figure 6, the adrenal gland coefficient was significantly increased (*p* < 0.05), and the thymus and spleen coefficients were significantly decreased (*p* < 0.05) in the model group compared with the normal group. The treatment with HLS significantly increased thymus and spleen coefficients (*p* < 0.05).

### Effects of HLS on Pathomorphology of the Hippocampus and Adrenal Gland in Depressive Rats

#### Effects of HLS on the Morphology and Number of Neurons in the CA3 Area of the Hippocampus

**FIGURE 7:**
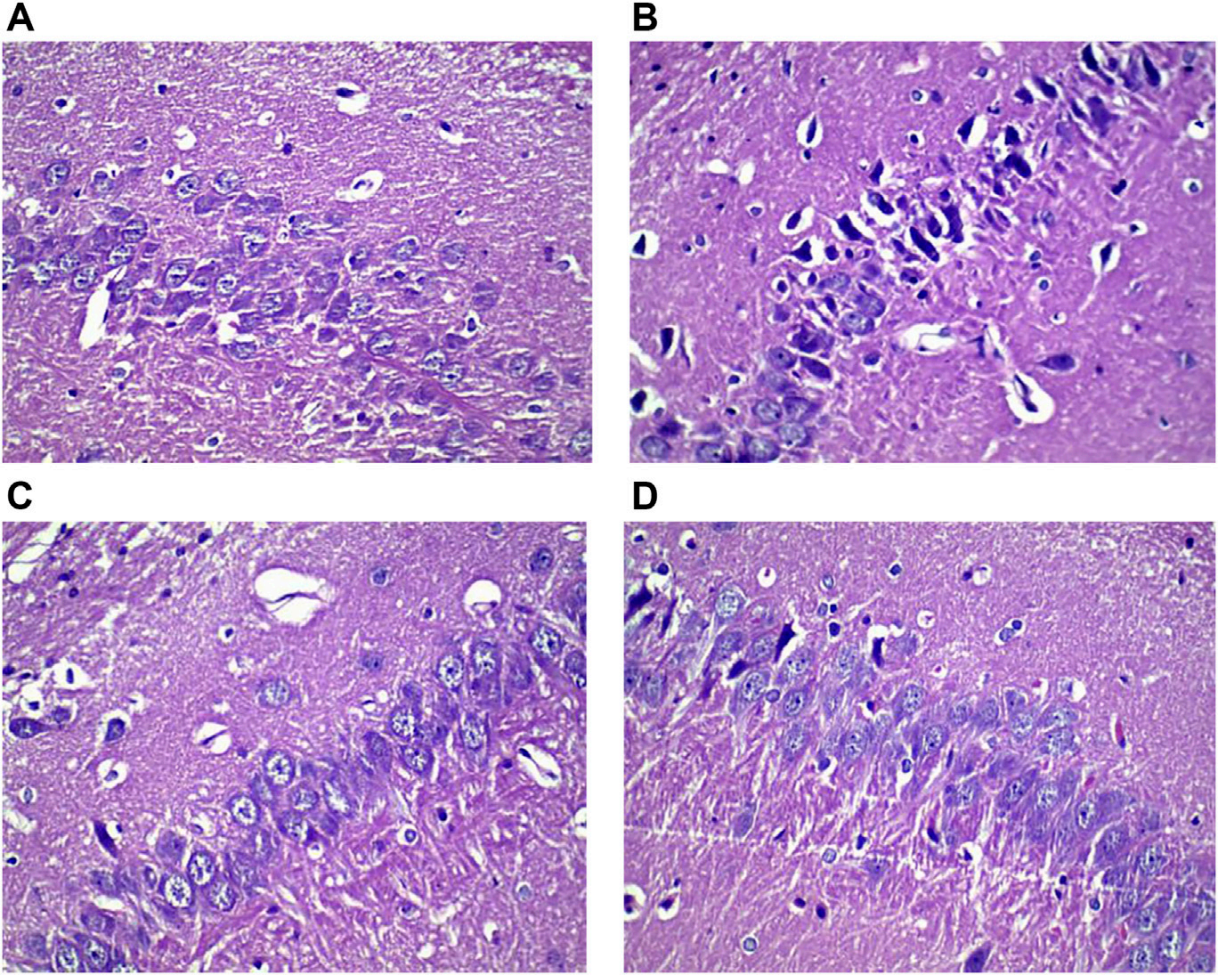
Histological images showing neurons in the CA3 area of the hippocampus of rats in different groups (HE staining, ×400). **(A)** Normal group, **(B)** model group, **(C)** sertraline group, and **(D)** HLS group.

**FIGURE 8:**
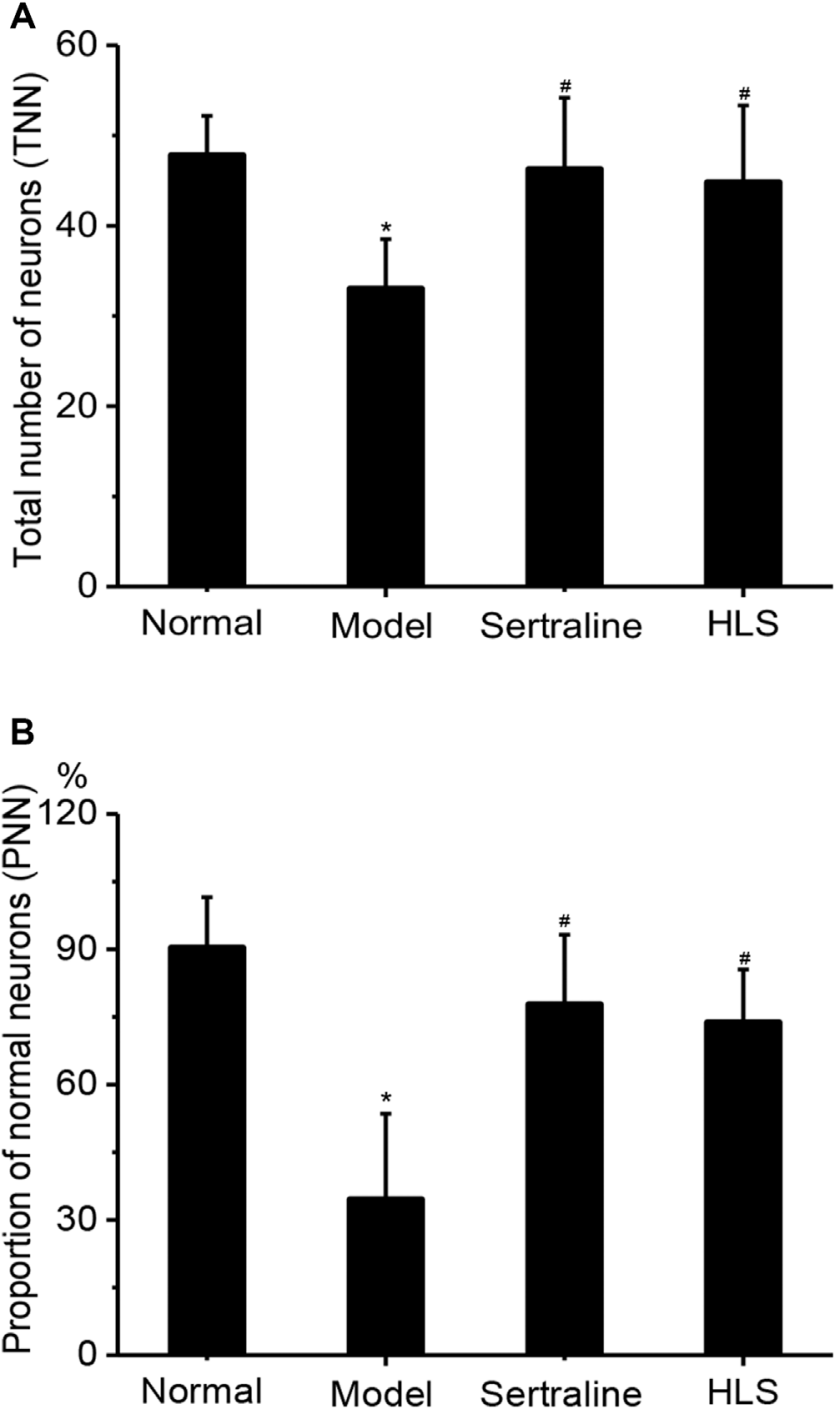
Effects of HLS on the total number of neurons and the proportion of normal neurons in the CA3 area of the hippocampus and the thickness of the adrenal cortex in rats (‾x ± s, *n* = 8). *p < 0.05 vs. normal control and ^*#*^ *p* < 0.05 vs. model. **(A)** Total number of neurons and **(B)** proportion of normal neurons.

The neurons in the CA3 area of the hippocampus of rats were orderly and densely arranged, with clearly visible nucleoli in the normal group. In contrast, the neurons in the CA3 area of the hippocampus of rats were sparsely arranged and showed increased intercellular space, nucleolus disappearance, and cytoplasm pyknosis in the model group (Figure 7). The total number of neurons and the proportion of normal neurons in the CA3 area of the hippocampus were significantly decreased in the model group compared with the normal group (*p* < 0.05). Compared with the model group, the total number of neurons and the proportion of normal neurons in the CA3 area of the hippocampus were significantly increased in the HLS groups (*p* < 0.05) (Figure 8).

#### Effects of HLS on the Thickness of the Adrenal Cortex and Pathomorphology of the Adrenal Gland

**FIGURE 9:**
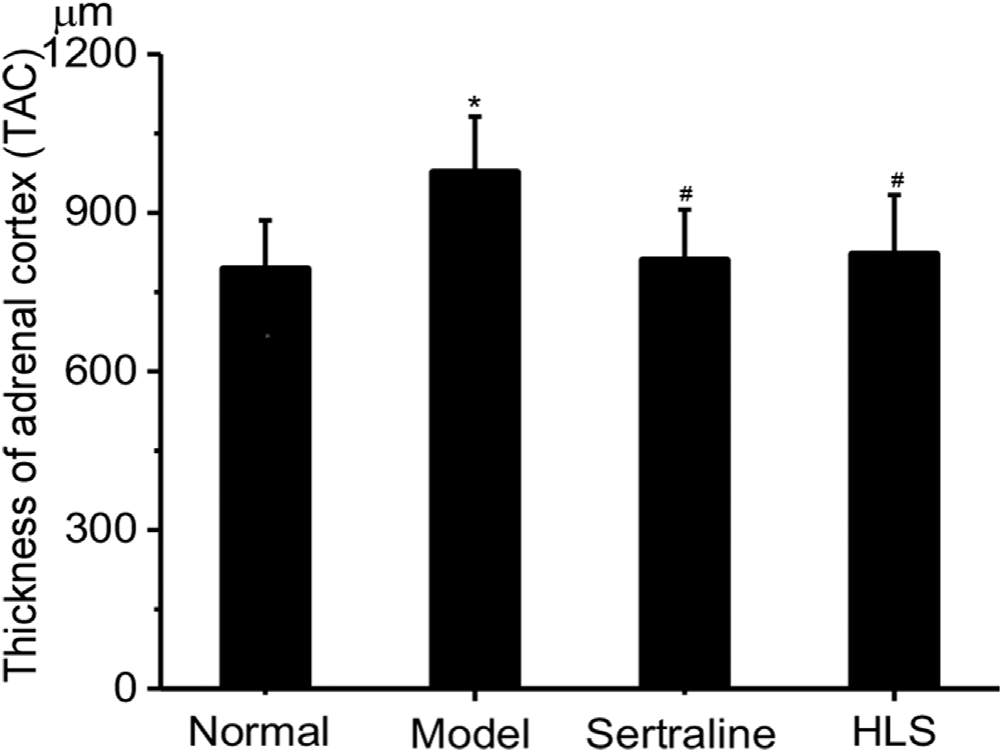
Effects of HLS on the thickness of the adrenal cortex in rats (‾x ± s, *n* = 8). *p < 0.05 vs. normal control; ^*#*^ *p* < 0.05 vs. model.

The adrenal glands of the rats had a clear structure and can be divided into two parts: adrenal cortex and medulla, and the adrenal cortex can be further divided into the following zones: zona glomerulosa, zona fasciculata, and zona reticularis in the normal control group. The adrenal cortex was hypertrophic in the model group. The thickness of the adrenal cortex was significantly larger in the model group than that in the normal group (*p* < 0.05), while treatment with HLS significantly decreased the thickness of the adrenal cortex compared with the model group (*p* < 0.05) (Figure 9).

### Contents of Active Ingredients

#### Content of 2,3,5,4′-Tetrahydroxy Stilbene-2-O-β-D-Glucoside in HLS

**FIGURE 10:**
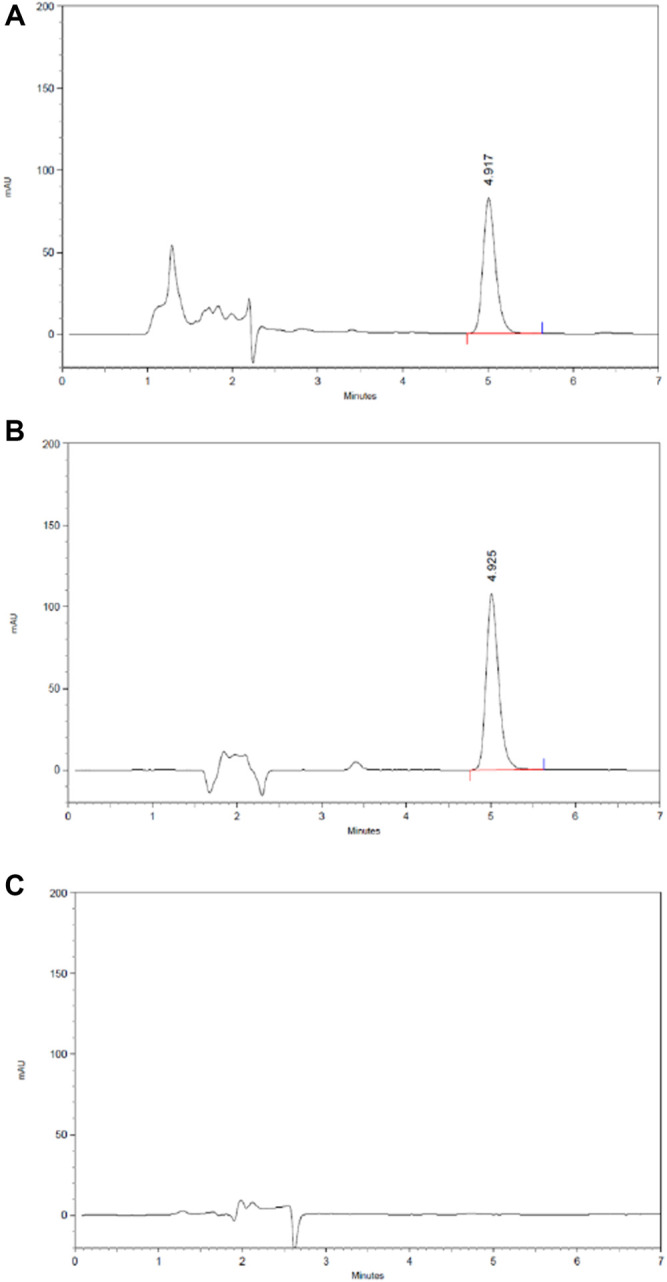
HPLC chromatography of the component of 2,3,5,4′-tetrahydroxy stilbene-2-O-β-D-glucoside in HLS. **(A)** HLS, **(B)** 2,3,5,4′-tetrahydroxy stilbene-2-O-β-D-glucoside, and **(C)** negative sample without polygoni multiflora radix praeparata.

The content of 2,3,5,4′-tetrahydroxy stilbene-2-Ο-β-D-glucoside in HLS is 3.85 mg/ml based on HPLC (Figure 10).

#### Content of Icariin in HLS

**FIGURE 11:**
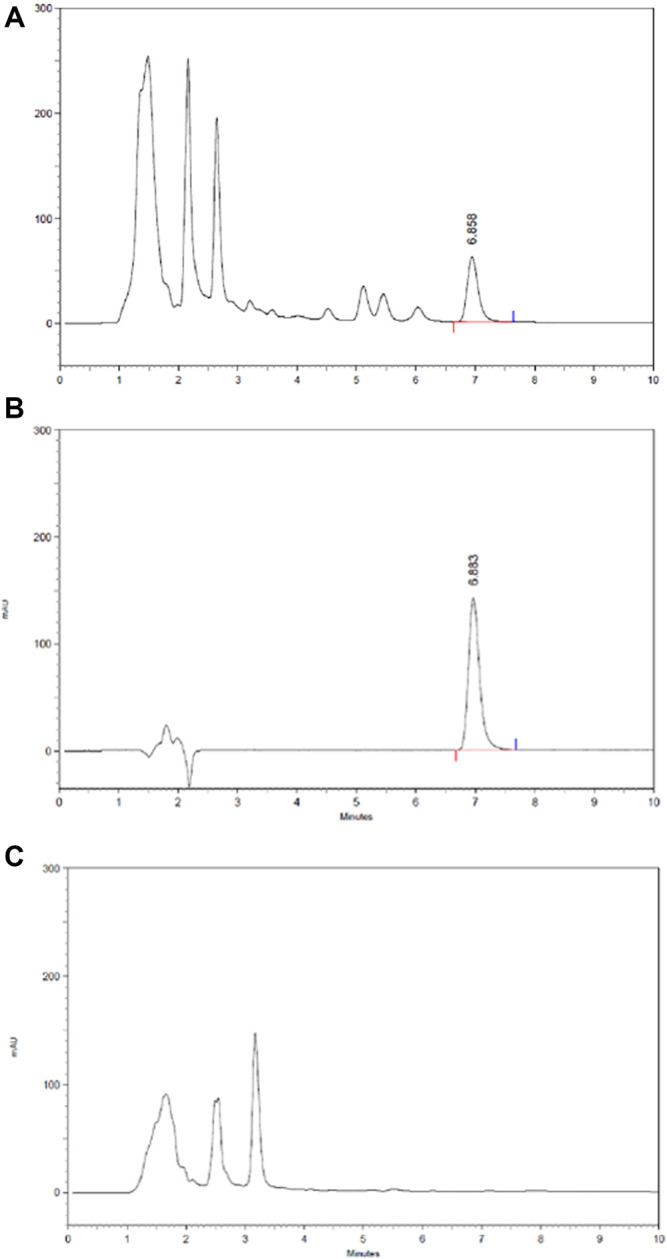
HPLC chromatography of components of icariin in HLS. **(A)** HLS, **(B)** icariin, and **(C)** negative sample without epimedii folium.

The content of icariin in HLS is 1.04 mg/ml based on HPLC (Figure 11).

#### Content of Saponins of Rhizoma Polygonati in HLS

The content of Saponins of Rhizoma Polygonati in HLS is 1.22 mg/ml determined by colorimetry.

#### Content of Astragaloside IV in HLS

**FIGURE 12:**
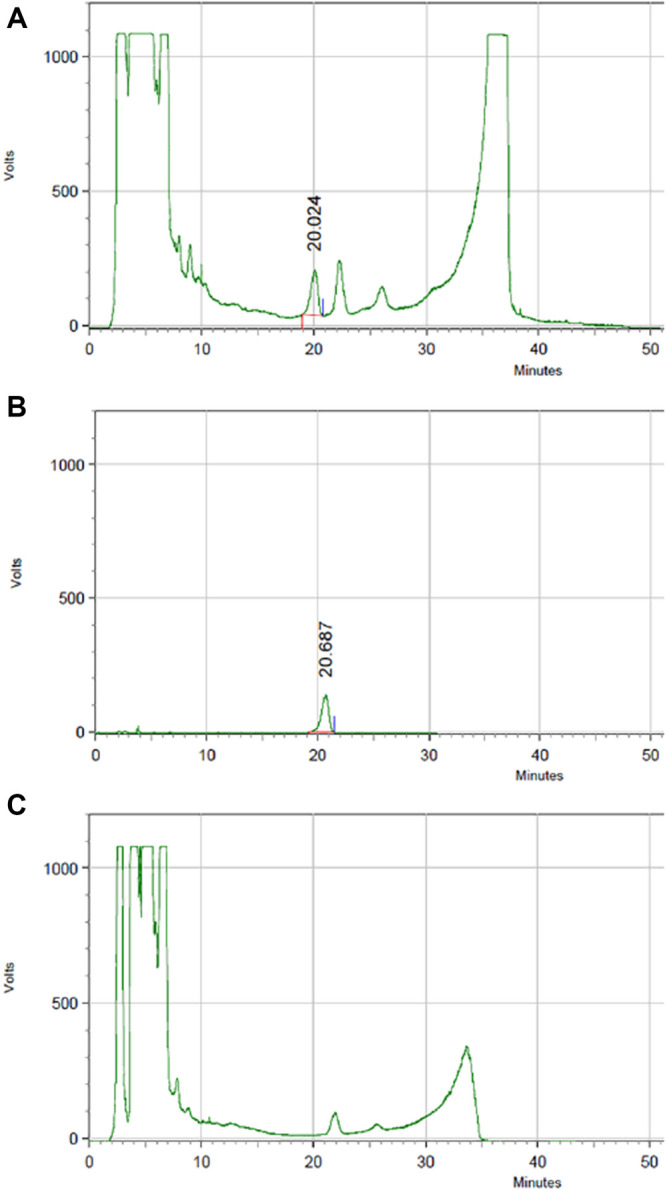
HPLC chromatography of the components of astragaloside IV in HLS. **(A)** HLS, **(B)** astragaloside IV, and **(C)** negative sample without astragali radix.

The content of astragaloside IV in HLS is 0.036 mg/ml based on HPLC (Figure 12).

#### Content of Tanshinone ⅡA in HLS

**FIGURE 13:**
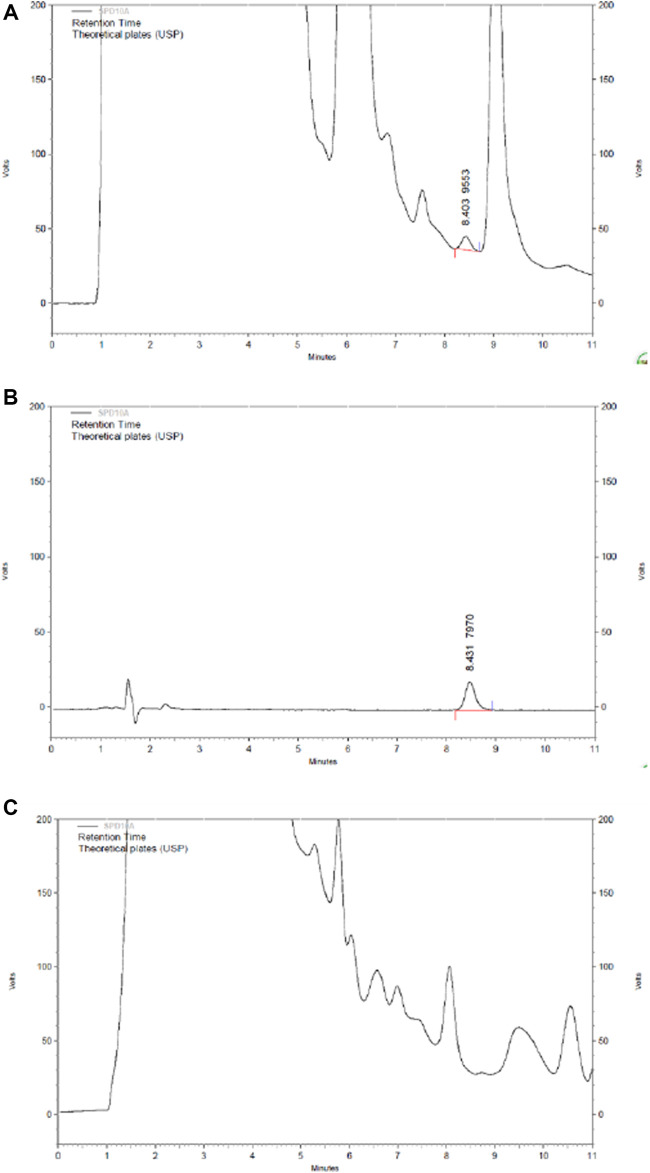
HPLC chromatography of components of tanshinone ⅡA in HLS. **(A)** HLS, **(B)** tanshinone ⅡA, and **(C)** negative sample without salviae miltiorrhizae radix et rhizoma.

The content of tanshinone ⅡA in HLS is 0.024 μg/ml based on HPLC (Figure 13).

## Discussion

Depression is a chronic, recurring, and life-threatening mental illness that is characterized by hypobulia, sluggishness of thinking, and persistent depression in spirits. Although the pathogenesis of depression is not clear until now, many disorder models of animals are successfully established to simulate the depressive situation. Also, they play a crucial part in scientific screening and evaluation of depression, including behavioral despair tests, learned helplessness models, CUMS models, and separation models, as well as neurobiochemical models (Willner, 1984). In this study, we mainly chose stress models: FST, TST, CUMS, and separation.

The behaviors of mice in FST and TST are similar to some components of depression in clinical, so they have been widely used for screening and evaluation of antidepressants (Chang et al., 2006; Mcarthur and Borsini, 2006; Chiba et al., 2012). The study showed that the HLS group had a shorter immobility time in the FST and TST compared to the control group with a significant difference, and the results of the locomotor activity test showed HLS had no central stimulating effect (i.e., the shortened immobility time was not due to central stimulation) (Zhou et al., 2012); overall, HLS has a potential effect on antidepression, but it is not associated with central stimulation.

In consideration of the validity of the chronic depressive model, presently, a rat model of CUMS-induced depression was established, which can truly simulate the environmental causes of depression and well respond to classic antidepressant treatment (Chang et al., 2006). Bodyweight and the open field score were used in this model to evaluate the effect of HLS in improving depressive symptoms in rats. The results from behavior determination showed that HLS could obviously increase the bodyweight of rats with CUMS-induced depression and enhance the activity of depressive rats after a 3-week treatment. These findings suggested that HLS could improve the depressive behaviors of rats (Mcarthur and Borsini, 2006).

The results from neurotransmitter content determination showed that 5-HT, DA, and NE levels were significantly decreased in the hippocampus but increased in the serum of rats with CUMS-induced depression. After treatment with HLS, the 5-HT, DA, and NE levels were significantly increased in the hippocampus and dropped to nearly normal in serum. These findings suggested that the antidepressive effect of HLS was possibly related to the regulation of the content of monoamine neurotransmitters in the body (Chiba et al., 2012; Liu et al., 2012; Zhou et al., 2012). It was consistent with the reports of Liu and Cao (Liu MY. et al., 2017; Cao and Li, 2017; Li et al., 2018). According to the monoamine neurotransmitter hypothesis, the pathogenesis of depression may be related to the decreased release of monoamine neurotransmitters by central synapses. Moreover, the concentration of 5-HT in the blood may be associated with its different processes of synthesis, storage, release, and degradation (Shuttleworth and Brien, 2001). In general, our study implicated that monoamine neurotransmitters can be normalized by HLS.

The CORT and ACTH levels in blood and the adrenal gland coefficient are increased in depressive rats. The treatment with HLS significantly decreased the CORT and ACTH levels and had a tendency to decrease the adrenal gland coefficient (*p* = 0.093). There have been many other similar reports in the literature (Zhang JM. et al., 2010; Uschold-Schmidt et al., 2012; Yi et al., 2012). In addition, other hormones including β-EP are also associated with stress (Hegadoren et al., 2009; Yim et al., 2010; Sadeghian et al., 2012). Furthermore, the β-EP level in blood was higher in depressive rats. Clinically, the increased levels of β-EP have also been reported in patients with depression (Tong and Li, 2008). In stressful conditions, sustained excitement of the sympathetic-adrenal medulla system and HPA axis can cause persistently increased β-EP level (Li et al., 2008; Tong and Li, 2008) and obvious damage to the body (Ahsin et al., 2009). In the present study, after treatment with HLS, the content of β-EP dropped to a normal level, indicating that HLS can simultaneously regulate the nervous and endocrine functions in depressive rats.

In addition, depression increases the release of the excitatory neurotransmitter glutamate in the hippocampus (He et al., 2009), thus resulting in the increase in NO synthesis, eventually leading to hippocampal neuronal apoptosis (Xiao and Chen, 2008) and adversely affecting hippocampal function (Chen and Dong, 2008; Zhang Y. et al., 2010; Dranovsky and Lenonardo, 2012). The present study showed that HLS had significant protective effects on hippocampal neurons and could significantly increase the proportion of surviving hippocampal neurons.

The thymus and spleen coefficients decreased in depressive rats, while treatment with HLS could reverse such changes. The decrease in the weight of two immune organs, the thymus and the spleen, may be related to the decrease in the immune function of depressive rats (Chen et al., 2005). Some scholars (Blume et al., 2011) also proposed that lymphocyte proliferation response is significantly inhibited in rats with chronic stress-induced depression. Therefore, we speculated that the therapeutical effect of HLS is in part related to enhancing the immune system of the rats.

To determine the content of the main antidepressant ingredients of HLS, we searched the relevant literature extensively. Based on the literature, we concluded that 2,3,5,4′-tetrahydroxy stilbene-2-Ο-β-D-glucoside in polygoni multiflora radix praeparata has immunity enhancing and neuroprotective effects (Miller, 2010), icariin in epimedii folium can upregulate the 5-HT receptor expression in the brain of depressive animals (Zhong et al., 2005), saponins of rhizoma polygonati can regulate the depressive behavior and the hippocampal 5-HT1AR/Camp/PKA signaling pathway in rats with depression (Gu and Lu, 2008), astragaloside has immunity enhancing and anti-inflammatory effects (Lin et al., 2011), and tanshinone IIA has a protective effect on nerve cells (Zhao et al., 2006); thus, we concluded that these compounds may all have some antidepressive effect. We measured the content of these compounds in HLS, and the results revealed that the contents of 2,3,5,4′-tetrahydroxy stilbene-2-Ο-β-D-glucoside, icariin, and saponins of rhizoma polygonati are high in HLS, thus demonstrating antidepressive effect of HLS from the point of the content of the HLS ingredients.

## Conclusion

In this study, we confirmed the anti-depressive effects of HLS and discussed the potential mechanisms involved using a rat model of CUMS-induced depression. We found that HLS regulates 5-HT, DA, NE, CORT, ACTH, and β-EP levels. The mechanism of the effects may be associated with its regulation on the neuroendocrine level and HPA axis. It may also be related to the enhancement of immune function, but it remains to be further confirmed. Furthermore, our findings provided a theoretical basis for the future applications of HLS in clinical antidepressant prevention strategies.

## Data Availability

The raw data supporting the conclusions of this article will be made available by the authors, without undue reservation.
